# Prognostic value of continuous EEG monitoring during therapeutic hypothermia after cardiac arrest

**DOI:** 10.1186/cc9276

**Published:** 2010-09-29

**Authors:** Andrea O Rossetti, Luis A Urbano, Frederik Delodder, Peter W Kaplan, Mauro Oddo

**Affiliations:** 1 Department of Clinical Neurosciences, Lausanne University Hospital and Faculty of Biology and Medicine, BH-07, Rue du Bugnon 46, CHUV, 1011 Lausanne, Switzerland; 2 Department of Intensive Care Medicine, Lausanne University Hospital and Faculty of Biology and Medicine, BH-08, Rue du Bugnon 46, CHUV, 1011 Lausanne, Switzerland; 3 Department of Neurology, Johns Hopkins Bayview Medical Center, 4940 Eastern Avenue, Baltimore, Maryland 21224, USA

## Abstract

**Introduction:**

Continuous EEG (cEEG) is increasingly used to monitor brain function in neuro-ICU patients. However, its value in patients with coma after cardiac arrest (CA), particularly in the setting of therapeutic hypothermia (TH), is only beginning to be elucidated. The aim of this study was to examine whether cEEG performed during TH may predict outcome.

**Methods:**

From April 2009 to April 2010, we prospectively studied 34 consecutive comatose patients treated with TH after CA who were monitored with cEEG, initiated during hypothermia and maintained after rewarming. EEG background reactivity to painful stimulation was tested. We analyzed the association between cEEG findings and neurologic outcome, assessed at 2 months with the Glasgow-Pittsburgh Cerebral Performance Categories (CPC).

**Results:**

Continuous EEG recording was started 12 ± 6 hours after CA and lasted 30 ± 11 hours. Nonreactive cEEG background (12 of 15 (75%) among nonsurvivors versus none of 19 (0) survivors; *P *< 0.001) and prolonged discontinuous "burst-suppression" activity (11 of 15 (73%) versus none of 19; *P *< 0.001) were significantly associated with mortality. EEG seizures with absent background reactivity also differed significantly (seven of 15 (47%) versus none of 12 (0); *P *= 0.001). In patients with nonreactive background or seizures/epileptiform discharges on cEEG, no improvement was seen after TH. Nonreactive cEEG background during TH had a positive predictive value of 100% (95% confidence interval (CI), 74 to 100%) and a false-positive rate of 0 (95% CI, 0 to 18%) for mortality. All survivors had cEEG background reactivity, and the majority of them (14 (74%) of 19) had a favorable outcome (CPC 1 or 2).

**Conclusions:**

Continuous EEG monitoring showing a nonreactive or discontinuous background during TH is strongly associated with unfavorable outcome in patients with coma after CA. These data warrant larger studies to confirm the value of continuous EEG monitoring in predicting prognosis after CA and TH.

## Introduction

Therapeutic hypothermia (TH) improves outcome in comatose survivors of cardiac arrest (CA) [1-3]. TH also alters the predictive value of neurologic prognostication in patients with postanoxic coma [4]. We and others recently demonstrated that, compared with previous studies performed before the introduction of TH [5], neurologic examination performed at 72 hours may be unreliable to predict outcome after CA, and that standard EEG may significantly improve prognostication at this time [6,7].

Continuous EEG monitoring (cEEG) provides important information regarding brain function, particularly in comatose patients [8,9], and is increasingly used to monitor early on-line changes of cerebral electrophysiology at the bedside in critically ill patients. Only a few studies have evaluated the role of cEEG performed during TH in the early phase of postresuscitation care. These studies, however, either included pediatric populations only [10] or were focused primarily on the prevalence of postanoxic seizures [11]. However, the exact prognostic value of cEEG findings during TH in patients with postanoxic coma has not been investigated. In this prospective study, we sought to examine the relation between cEEG findings during TH and outcome in comatose survivors of CA. We primarily tested the hypothesis that the type and reactivity of cEEG background during TH may reliably predict patient prognosis.

## Materials and methods

### Patients

We prospectively studied consecutive comatose adult patients (older than 16 years) admitted from April 2009 to April 2010 to the medicosurgical intensive care unit (ICU) of the University Hospital of Lausanne, who were treated with TH after successful resuscitation from CA and were monitored with cEEG, initiated during hypothermia. Approval for the study was obtained by the local Institutional Review Board with waiver of informed consent, because cEEG was part of standard patient care. All patients were resuscitated according to current recommendations [2] and treated with mild TH to 33°C for 24 hours. Therapeutic hypothermia was started immediately after admission to the emergency department and was applied by using a cooling technique combining the administration of intravenous ice-cold fluids and the application of a surface cooling device (Arctic Sun System; Medivance, Louisville, CO, USA), according to the protocol in use in our institution [6,12]. Midazolam (0.1 mg/kg/h) and fentanyl (1.5 μg/kg/h) were given for sedation-analgesia, and vecuronium (0.1 mg/kg boluses) was administered to control shivering.

### Continuous EEG data

Video-cEEG (Viasys Neurocare, Madison, WI, USA) was started as soon as possible after ICU admission and during TH, by using nine to 21 electrodes arranged according to the international 10-20 system, and was maintained up to at least 6 hours after rewarming. Background reactivity on cEEG was tested with repetitive auditory, visual, and nociceptive stimulations performed by an experienced neurologist during and after TH, as described in our previous study [6]. Within 4 hours after the end of cEEG, all recordings were interpreted by two EEG-certified neurologists; cEEG background reactivity was considered present if cerebral electrical activity of at least 10 μV (regardless of frequency range) was observed, and EEG background showed any clear and reproducible change in amplitude or frequency on simulation, excluding "stimulus-induced rhythmic, periodic, or irritative discharges" (SIRPIDS) or induction of muscle artifact alone. Stimulation and EEG background activity were assessed in all patients after at least 12 hours after the start of TH (that is, during the maintenance phase of TH) and within 24 hours from CA: thus, EEG background reactivity was tested before the 72-hour delay recommended by the American Academy of Neurology [5]. EEG background interrupted by flat periods was labeled as "discontinuous" (in this setting, also known as "burst-suppression") if this pattern was found over the whole recording. Repetitive or rhythmic, focal or generalized spikes, sharp waves, spike and waves, or rhythmic waves evolving in amplitude, frequency, or field were categorized as "epileptiform," as detailed in our previous studies [6,13,14].

### Additional standard assessments and treatment

The following investigations were performed shortly after rewarming, at least 36 hours after CA, at a patient core temperature >35°C and off sedation, as previously reported [6]: repeated neurologic examination, a standard (30 minute) EEG with the previously mentioned stimulations, and cortical somatosensory evoked potentials (SSEPs). Patients with EEG evidence of status epilepticus were treated with intravenous antiepileptic drugs (including levetiracetam, midazolam, valproate, or propofol for at least 24 hours), as reported in our previous study [14]. Treatment was discontinued if no clinical improvement was noted after at least 72 hours, together with incomplete recovery of all brainstem reflexes (pupillary, oculocephalic, corneal), and/or bilaterally absent cortical response of SSEPs, in accordance with current recommendations [5]. Physicians were not blinded to the cEEG results; however, cEEG findings were not used to guide therapy or to decide withdrawal of care.

### Data collection

Baseline demographics, including type of CA (ventricular fibrillation (VF) versus non-VF, including asystole and pulseless electrical activity), time from CA to return of spontaneous circulation (ROSC), etiology of CA (cardiac versus noncardiac), and time from CA to temperature target of 33°C were prospectively collected. The following cEEG data were recorded during TH and included in the analysis: presence/absence of background reactivity, presence/absence of discontinuous EEG background, and presence/absence of epileptiform abnormalities.

### Outcome assessment

In-hospital mortality was used as primary outcome. Neurologic outcome was assessed at 2 months by review of the computerized database of our hospital or a phone interview, and categorized according to the Glasgow-Pittsburgh Cerebral Performance Categories (CPC), in which 1 = good recovery, 2 = moderate disability, 3 = severe disability with dependency for daily-life activity, 4 = vegetative state, and 5 = death [15], and outcome was dichotomized as good (CPC 1 and 2) versus poor (CPC 3 to 5).

### Statistical analysis

Quantitative parameters are reported as median and range, and dichotomous variables, as number and percentage. Two-sided *t *tests, Fisher Exact, and Mann-Whitney *U *tests were used as needed. Significance was assumed at a level of *P *< 0.01, applying conservative analysis for multiple comparisons between variables (Bonferroni corrections, with five tests). Positive (PPV) and negative (NPV) predictive values for mortality and false-positive rates (FPR; 1-specificity) were calculated by using a binomial 95% CI. Area under the receiver operating characteristic (ROC) curve was used to assess the predictive values for mortality, and comparisons were analyzed by using nonparametric tests. Calculations were performed with Stata software, version 9 (College Station, TX, USA).

## Results

### Patients

We studied 34 comatose CA survivors treated with TH for 24 hours and monitored with cEEG during TH. Mean patient age was 61 ± 13 years; median time from CA to ROSC was 20 (interquartile range, 10 to 30) minutes; mean time from CA to cEEG recording was 12 ± 6 hours; cEEG lasted a mean of 30 ± 11 hours. No complication related to the cEEG was observed; shivering, muscle, or electrode artifacts were transient and did not interfere with interpretation.

### Relation between baseline clinical variables and outcome

At 2 months, 15 patients died, and 19 patients survived. The majority of survivors (14 (74%) of 19 patients) had a good outcome (*n *= 8 with CPC 1; *n *= 6 with CPC 2), whereas the remaining five patients had CPC 3. No patient remained in a vegetative state. Baseline demographic variables, including gender, initial arrest rhythm, CA etiology, and time from CA to ROSC were comparable between survivors and nonsurvivors (Table 1).

**Table 1:** Patient baseline characteristics in survivors versus nonsurvivors

	Survivors (*n *= 19)	Nonsurvivors (*n *= 15)
Female gender, number (%)	6 (32%)	3 (20%)
Median age, years (range)	62 (35-84)	64 (32-73)
Initial CA rhythm ventricular fibrillation, number (%)	14 (73%)	10 (67%)
CA of cardiac etiology, number (%)	16 (84%)	11 (73%)
Median time from CA to ROSC, minutes (range)	20 (5-40)	22 (8-180)

CA, cardiac arrest; ROSC, return of spontaneous circulation.

### Early continuous EEG findings and outcome

**Figure 1:**
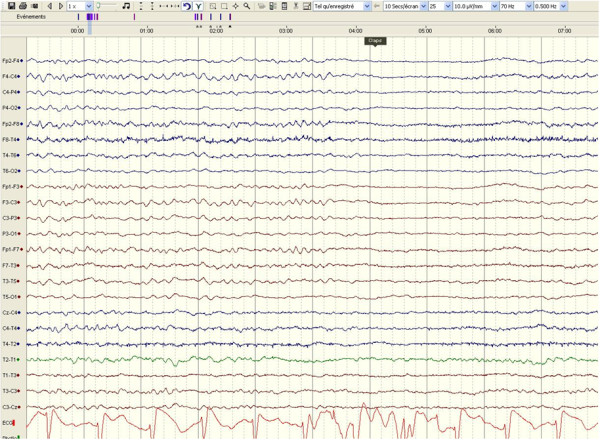
**EEG recording performed during therapeutic hypothermia from one representative patient who had a good outcome (Cerebral Performance Category 1 at 2 months)**. EEG shows a reactive EEG background activity to sound ("claps"); recording, 30 mm/sec, 10 μV/mm.

**Figure 2:**
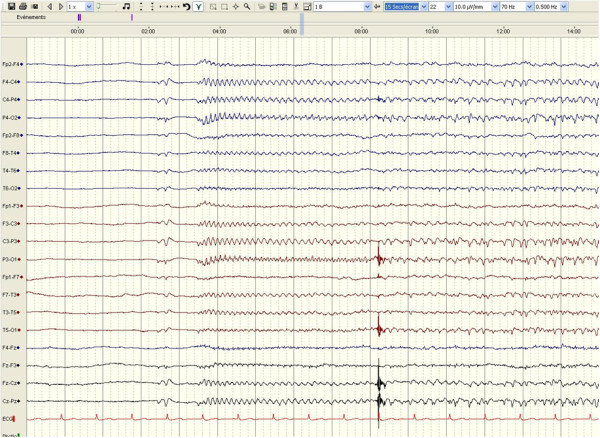
**EEG recording performed during therapeutic hypothermia from one representative patient who died**. EEG shows discontinuous EEG background activity, alternating with generalized, electrical seizures ("seizure-suppression pattern"). EEG was nonreactive to painful stimuli; recording, 20 mm/sec, 10 μV/mm.

Representative examples of EEG recordings during TH are given in Figure 1, showing one patient with a reactive cEEG background who eventually had a good recovery (Figure 1) and another patient with a persistent discontinuous EEG background activity alternating with generalized, electrical seizures ("seizure-suppression pattern"), who eventually died (Figure 2).

The association between outcome and cEEG findings during TH is shown in Table 2. After adjusting for multiple comparisons, nonreactive EEG background, persistent discontinuous EEG pattern, and presence of seizures/epileptiform discharges were strongly associated with mortality. Importantly, all patients with epileptiform abnormalities or persistent discontinuous EEG background or both also showed absent EEG reactivity. Predictive values for mortality for these three cEEG features, as well as SSEPs, are shown in Table 3. Despite relatively wide confidence intervals due to the small sample size, the positive predictive value (PPV) was 100%, and the false-positive rate (FPR) was 0, thus indicating excellent prognostic value for early cEEG features. Of note, compared with patients with a reactive cEEG background, those with nonreactive cEEG backgrounds received similar weight-adjusted doses of midazolam (*P *= 0.49; *t *test) and fentanyl (*P *= 0.33; *t *test).

**Table 2:** Continuous EEG characteristics in survivors versus nonsurvivors

	Survivors (*n *= 19)	Nonsurvivors (*n *= 15)	*P *value (test)
Time from CA to initiation of cEEG, hours (range)	16 (3-23)	10 (1-21)	0.11 (U)
Median cEEG duration, hours (range)	26 (19-48)	26 (22-66)	0.17 (U)
Nonreactive cEEG background, number (%)	0 (0)	12 (75%)	<0.001 (Fisher)
Prolonged discontinuous activity ("burst-suppression"), number (%)	0 (0)	11 (73%)	<0.001 (Fisher)
EEG seizures or epileptiform discharges, number (%)	0 (0)	7 (47%)	0.001 (Fisher)

CA, cardiac arrest.

**Table 3:** Prognostic predictive value of continuous EEG (30-day mortality)

	PPV	NPV	FPR
Nonreactive background	1.00 (0.74-1.00)	0.83 (0.65-0.97)	0 (0-0.18)
Prolonged discontinuous activity ("burst-suppression")	1.00 (0.71-1.00)	0.86 (0.61-0.95)	0 (0-0.18)
Seizures/epileptiform discharges	1.00 (0.59-1.00)	0.70 (0.50-0.86)	0 (0-0.18)
Bilaterally absent SSEPs	1.00 (0.48-1.00)	0.70 (0.50-0.86)	0 (0-0.18)

FPR, false-positive rate; NPV, negative predictive value; PPV, positive predictive value; SSEPs, somatosensory evoked potentials.

### Association between outcome and neurologic and electrophysiological examinations at 72 hours

**Figure 3:**
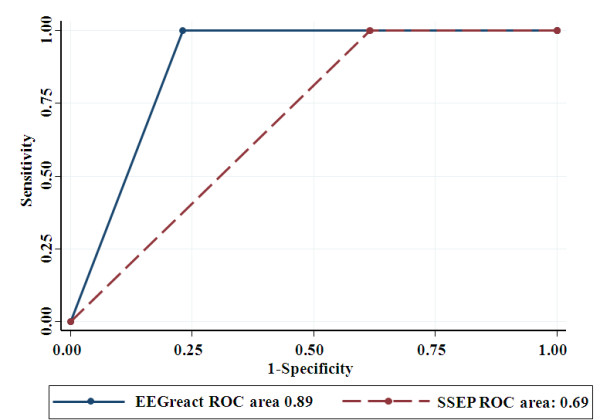
**Area under the receiver operating characteristic (ROC) curve for mortality prediction of cEEG reactivity (performed during therapeutic hypothermia, blue line) and of somatosensory evoked potentials (SSEPs, performed in normothermic conditions, red line)**. Continuous EEG yielded better prediction than SSEPs (ROC area, 0.88 versus 0.69; *P *= 0.006).

Neurologic examination and SSEPs were performed at 72 hours in normothermic conditions, as *per protocol *at our institution and according to actual recommendations [5]. All nonsurvivors with absent cEEG reactive background during TH also had absent SSEPs at 72 hours. Although the PPV for mortality of absent cEEG-reactive background and bilaterally absent SSEPs was 1.00, the NPV of cEEG was higher than that of SSEP (0.83 versus 0.70; Table 3). In addition, when using the area under the ROC curve (Figure 3), cEEG reactivity yielded better prediction than did SSEP, with a statistically significant difference in the predictive ability in favor of EEG background reactivity over SSEPs (0.88 versus 0.69; *P *= 0.006).

Incomplete recovery of brainstem reflexes (pupillary, oculocephalic, corneal) and absent or extension motor reaction to pain also differed among survivors and nonsurvivors (three of 19 versus 11 of 15, and three of 19 versus 15 of 15, respectively); however, the false-positive rate was greater than zero for both, confirming that neurologic examination alone may not be reliable in predicting the outcome after CA and TH.

### Postanoxic seizures and epileptiform discharges

The total number of patients with epileptiform EEG features during the entire study period was eight (26%) of 34. Five had generalized electrographic seizures alternating with diffuse suppression ("seizure-suppression" pattern), and two had generalized, sustained periodic epileptiform discharges (G-PEDs), again alternating with generalized background suppressions. One patient had delayed seizures that became apparent only after TH and rewarming. None of the seven patients with early (that is, during TH) epileptiform abnormalities showed a significant improvement on the standard EEG performed after TH in normothermic conditions. Furthermore, all had a nonreactive EEG background and died.

In contrast, in the single patient with delayed (that is, after TH, at normothermia) postanoxic seizures, cEEG became diffusely epileptiform with multifocal myoclonia only after weaning of sedation: of note, cEEG background remained reactive despite epileptiform activity, and the patient regained consciousness and survived.

## Discussion

The main results of this single-center prospective study can be summarized as follows: (1) absent EEG background reactivity observed during the maintenance phase of TH appeared to be strongly associated with poor outcome in patients with coma after CA; (2) all patients in whom cEEG showed background reactivity to painful stimuli survived, and the large majority (74%) awoke and had a favorable outcome; (3) persistent discontinuous background and the presence of seizures or epileptiform discharges on cEEG were also strong risk factors for poor outcome; (4) nonreactive cEEG background yielded a significantly better prognostic value than SSEPs, mostly because of a higher negative predictive value; (5) EEG reactivity to painful stimulation did not seem to be affected by TH, because all patients with absent background reactivity during TH had similar findings on the EEG performed in normothermic conditions, and it was not influenced by sedation-analgesia.

To our knowledge, this is the first clinical study showing that nonreactive EEG background activity during TH is an early predictor of poor outcome in patients with postanoxic coma. Before TH became a widely used treatment of hypoxic/ischemic encephalopathy, diffuse EEG background suppression below 20 μV, burst-suppression with generalized epileptiform activity, or generalized periodic complexes on a flat background have been associated with poor outcome [16,17]. This was recently confirmed by our group in patients treated with TH, in whom standard EEG was performed at the end of treatment in normothermic conditions [6]. Moreover, prolonged epileptiform EEG features are independently correlated with mortality after postanoxic coma [13], in patients assessed both after [6] and during [10,13] TH. However, none of these studies formally addressed the predictive value of any of the EEG findings during TH or compared the value of EEG with that of neurologic examination or SSEPs, the latter being regarded as reliable predictors of poor prognosis [5]. We have recently shown that background reactivity performed after TH in normothermic conditions is a strong outcome predictor of postanoxic coma [6], and thus undertook this study to examine the prognostic value of EEG background performed during TH in the early phase after CA. Our present findings confirm our previous study and indeed seem to suggest that reactive background on cEEG has a strong prognostic predictive value, even when monitoring is performed during TH. They also suggest that background reactivity is not significantly influenced by core temperature or by sedation. After earlier reports on favorable outcome for patients showing continuous amplitude-integrated EEG after TH [18], a recent study on 30 patients showed that quantitative EEG features during TH (burst-suppression ratio, response entropy, state entropy) were significantly associated with long-term functional outcome [19]. Although our results are in line with these findings, we add important concomitant clinical information and describe a much easier approach for EEG interpretation, without the need for more-complicated and not easily available software analysis.

Although our study was not primarily focused on the epidemiology of postanoxic seizures, this issue deserves further discussion. Previous studies reported a variable prevalence of postanoxic seizures from 10% [11] to 47% [10]. We observed a 21% prevalence (seven of 34 patients) of epileptiform abnormalities during TH, of whom five patients (15% of the entire cohort) had sustained EEG seizures. Because mild hypothermia and sedation (midazolam in our study) have antiepileptic action, the occurrence of electrical seizures during TH may reflect more-severe and diffuse brain injury. This might explain why none of the seven patients with seizures during TH survived, in line with previous observations [11]. In contrast, it appears that seizures occurring only at the end of TH, after rewarming and off sedation, carry a better prognosis, possibly because brain injury is less severe (thus they are effectively treated with induced hypothermia and sedatives). Indeed, one patient in our cohort, treated for status epilepticus that developed after TH, survived. Altogether, these data underline the value of early cEEG for the treatment of comatose CA patients treated with TH.

### Study limitations

This study has several limitations. First, the sample size is limited; thus our results are to be considered preliminary and will need further confirmation by other groups and larger studies. However, for this reason, we applied conservative statistical corrections for multiple comparisons (Bonferroni). Second, it was a single-center study, thus data cannot be generalized. Some subjectivity may also be related to the scoring of EEG reactivity; however, we used the same method described in our recent report, which included more than 100 patients. Time from CA to initiation of cEEG did not differ significantly between survivors and nonsurvivors (Table 2); thus it is unlikely that timing of cEEG affected the predictive value of the test. Finally, because the cEEG was interpreted before knowing final patient prognosis, it is unlikely that it influenced outcome. Furthermore, although clinicians were aware of cEEG results, EEG findings (both during TH and at normothermia) were not used to guide therapy or decisions for withdrawal of care; thus we believe that this contributed to minimize the so-called "self-fulfilling prophecy" phenomenon [6].

## Conclusions

Continuous EEG background abnormalities during TH seem to be strongly associated with outcome after CA and appear to yield excellent point estimates for positive predictive values and false-positive rates for mortality. Our data suggest that continuous EEG may be of value in predicting outcome after CA and TH. Additional larger prospective studies are needed to confirm our findings and to verify further whether continuous EEG can be helpful for the prognostic assessment of postanoxic coma.

## Key messages

• The results of this single-center study show that the presence of background reactivity on continuous EEG monitoring (cEEG) performed during therapeutic hypothermia is associated with 30-day survival and favorable neurologic outcome after cardiac arrest.

• Our preliminary data suggest that nonreactive EEG background carries a dismal outcome and is 100% predictive of mortality in comatose cardiac-arrest patients.

• Early cEEG findings appear to have a significantly better predictive value than somatosensory evoked potentials performed after TH.

• Additional larger prospective studies are needed to confirm whether continuous EEG may be a helpful tool for the prognostic assessment of postanoxic coma.

## Abbreviations

CA: cardiac arrest; cEEG: continuous electroencephalography; CPC: Glasgow-Pittsburgh Cerebral Performance Categories; EEG: electroencephalography; FPR: false-positive rate; G-PEDS: generalized, sustained periodic epileptiform discharges; ICU: intensive care unit; NPV: negative predictive value; PPV: positive predictive value; ROC: receiver operating characteristic; ROSC: return of spontaneous circulation; SIRPIDS: stimulus induced rhythmic, periodic, or irritative discharges; SSEPs: somatosensory evoked potentials; TH: therapeutic hypothermia; VF: ventricular fibrillation.

## Competing interests

The authors declare that they have no competing interests.

## Authors' contributions

AOR conceived the study, collected the data, carried out part of the data analysis, and drafted the manuscript. LAU carried out part of the data analysis and drafted the manuscript. FD helped with data collection and study coordination and revised the manuscript. PWK revised the manuscript and gave important intellectual contributions. MO conceived the study, was responsible for study coordination, and revised and helped to draft the manuscript.
